# Improved therapy-success prediction with GSS estimated from clinical HIV-1 sequences

**DOI:** 10.7448/IAS.17.4.19743

**Published:** 2014-11-02

**Authors:** Alejandro Pironti, Nico Pfeifer, Rolf Kaiser, Hauke Walter, Thomas Lengauer

**Affiliations:** 1 Computational Biology and Applied Algorithmics, Max Planck Institute for Informatics, Saarbrücken, Germany; 2 Department of Clinical Virology, University of Cologne, Cologne, Germany; 3 Diagnostics, Medizinisches Infektiologiezentrum Berlin, Berlin, Germany

## Abstract

**Introduction:**

Rules-based HIV-1 drug-resistance interpretation (DRI) systems disregard many amino-acid positions of the drug's target protein. The aims of this study are (1) the development of a drug-resistance interpretation system that is based on HIV-1 sequences from clinical practice rather than hard-to-get phenotypes, and (2) the assessment of the benefit of taking all available amino-acid positions into account for DRI.

**Materials and Methods:**

A dataset containing 34,934 therapy-naïve and 30,520 drug-exposed HIV-1 pol sequences with treatment history was extracted from the EuResist database and the Los Alamos National Laboratory database. 2,550 therapy-change-episode baseline sequences (TCEB) were assigned to test set A. Test set B contains 1,084 TCEB from the HIVdb TCE repository. Sequences from patients absent in the test sets were used to train three linear support vector machines to produce scores that predict drug exposure pertaining to each of 20 antiretrovirals: the first one uses the full amino-acid sequences (DE_full_), the second one only considers IAS drug-resistance positions (DE_onlyIAS_), and the third one disregards IAS drug-resistance positions (DE_noIAS_). For performance comparison, test sets A and B were evaluated with DE_full_, DE_noIAS_, DE_onlyIAS_, geno2pheno_[resistance]_, HIVdb, ANRS, HIV-GRADE, and REGA. Clinically-validated cut-offs were used to convert the continuous output of the first four methods into susceptible-intermediate-resistant (SIR) predictions. With each method, a genetic susceptibility score (GSS) was calculated for each therapy episode in each test set by converting the SIR prediction for its compounds to integer: S=2, I=1, and R=0. The GSS were used to predict therapy success as defined by the EuResist standard datum definition. Statistical significance was assessed using a Wilcoxon signed-rank test.

**Results:**

A comparison of the therapy-success prediction performances among the different interpretation systems for test set A can be found in Table 1, while those for test set B are found in Figure 1. Therapy-success prediction of first-line therapies with DE_noIAS_ performed better than DE_onlyIAS_ (p<10–16).

**Conclusions:**

Therapy success prediction benefits from the consideration of all available mutations. The increase in performance was largest in first-line therapies with transmitted drug-resistance mutations.

**Figure 1:**
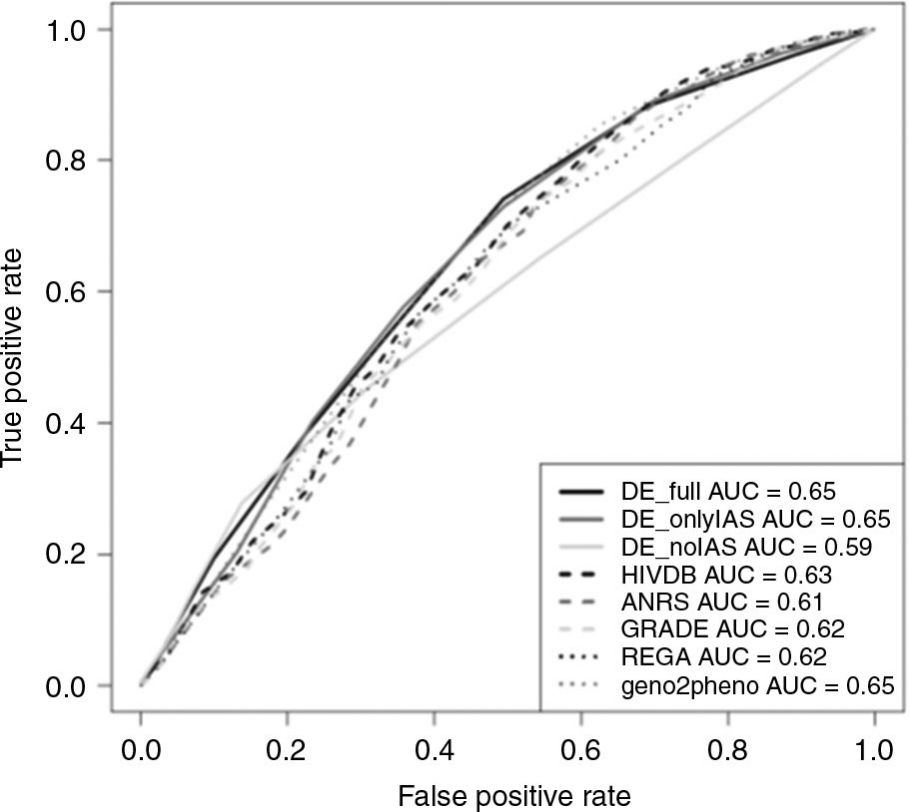
Performance comparison for therapy-success prediction in test set B.

**Table 1:** Performance comparison for therapy-success prediction in test set A, quantified via area under the receiver operating characteristic curve (AUC)

	DE_full_	DE_onlyIAS_	DE_noIAS_	HIVdb	ANRS	GRADE	REGA	geno2pheno_[resistance]_
All first-line therapies	0.54	0.52	0.6	0.51	0.51	0.5	0.52	0.53
First-line therapies with TDR	0.64	0.58	0.69	0.48	0.54	0.46	0.53	0.5
First-line therapies without TDR	0.51	0.5	0.58	0.5	0.5	0.5	0.51	0.52
Therapies on pretreated patients	0.68	0.69	0.59	0.68	0.67	0.69	0.69	0.69
All	0.67	0.67	0.65	0.66	0.65	0.66	0.66	0.66

